# Under-reporting of birth registrations in New South Wales, Australia

**DOI:** 10.1186/1471-2393-12-147

**Published:** 2012-12-12

**Authors:** Fenglian Xu, Elizabeth A Sullivan, Deborah A Black, Lisa R Jackson Pulver, Richard C Madden

**Affiliations:** 1 PRERU, University of New South Wales, Randwick, NSW, 2031, Australia; 2 Faculty of Health Sciences, University of Sydney, Lidcombe, NSW, 1825, Australia; 3 Muru Marri Indigenous Health Unit, School of Public Health & Community Medicine, University of New South Wales, Randwick, NSW, 2052, Australia

**Keywords:** Birth, Registration, Factor, Australia

## Abstract

**Background:**

To determine the rates of birth registration over a five-year period in New South Wales (NSW) and explore the factors associated with the rate of registration.

**Methods:**

This is a cross-sectional study using linked population databases. The study population included all births of NSW residents in NSW between 2001 and 2005.

**Results:**

Birth registration rates in NSW were 82.66% in the year of birth, 93.19% in the first year, 94.02% in the second, 94.56% in the third and 95.08% in the fourth year after birth. The non-registration of births was mainly associated with such factors as neonatal and postneonatal death (adjusted OR = 3.84, 95% CI: 3.23-4.57); being Indigenous (adjusted OR = 3.26, 95% CI: 3.10-3.43); maternal age <25 or >39 years (adjusted OR = 2.81, 95% CI: 2.72-2.90); low birthweight (<2,500 grams) (adjusted OR = 1.79, 95% CI: 1.69-1.90); living in remote areas (adjusted OR = 1.57, 95% CI: 1.52-1.63); being born after the first quarter of year (adjusted OR = 1.08-1.56, 95% CI between 1.03-1.12 and 1.49-1.64); mother having more pregnancies (adjusted OR = 1.85-7.29, 95% CI between1.78-1.93 and 6.87-7.73). Mothers who were born overseas were more likely to register their births than those born in Australia (adjusted OR = 0.72, 95% CI: 0.69-0.75). Multiple births were more likely to be registered than singleton births (adjusted OR = 0.84, 95% CI: 0.76-0.92). About one-third of the non-registrations of births in NSW were explained by the risk factors. The reasons for the remaining non-registrations need to be investigated.

**Conclusion:**

Of birth in NSW, 4.92% were not registered by the fourth year after birth.

## Background

In Australia, information on births is published annually by two organisations: the Australian Bureau of Statistics (ABS) and the Australian Institute of Health and Welfare (AIHW) National Perinatal Statistics Unit (NPSU) [1,2]. The ABS annually collates and publishes birth registration data that are collected by state and territory Registries of Births, Deaths and Marriages (RBDM). The NPSU compiles the National Perinatal Data Collection from Midwives Data Collections (MDC) in all jurisdictions. These data include all births of at least 20 weeks gestation or 400 grams birthweight [1]. Data are published annually in *Australia’s mothers and babies*[2].

The MDC consistently reports more live births than the RBDM by year of birth. In 2004, the MDC reported 255,286 live births which was 4.8% higher than the 243,680 live births in the birth registration report [2]. This pattern was evident in preceding years as well. This gap has been gradually increasing over time.

In New South Wales (NSW), the percentage difference between the MDC and RBDM was 2.4% in 2000, 3.9% in 2001, 4.3% in 2002, 5.5% in 2003 and 6.7% in 2004. There is also variation in the size of the gap among other states and territories in Australia. In 2004, the differences were 6.7% in NSW, 6.3% in Queensland, 4.8% in South Australia, 3.9% in Western Australia, 2.5% in Victoria, 0.9% in ACT, 0.8% in North Territory and 1.2% in Tasmania [2].

In order to determine the reasons for the discrepancies between the numbers of births collected by the NSW MDC and the NSW RBDM, birth registration rates were followed up for four years after birth using linked data. The birth registration rates and factors associated with the rates are described in this paper.

## Method

The study population included all live births of NSW residents recorded in the NSW MDC between January 2001 and December 2005. The birth records of the residents of other states and territories were excluded from the analysis.

Data is recorded in the MDC by either the midwife or medical staff. It includes all births in NSW of at least 20 weeks gestation or more than 400 grams birthweight, and includes maternal demographic factors, obstetric information and pregnancy outcomes. The NSW RBDM is recorded by the parents of the child and the forms are lodged with the Registrar of Births, Deaths and Marriages. It covers all births registered in NSW and includes demographic factors and some pregnancy outcomes. Study data were obtained from the NSW MDC and linked with the NSW RBDM. The births in the MDC were followed up until registration by the RBDM. The babies born from 2001 to 2005 were followed up until 2005. The births in 2001 had the longest follow-up period of four years.

For babies born in NSW from 1 January 2001 to 31 December 2005, there were 434,513 birth records, including 46 duplicate records, in the MDC and 405,366 birth records including 623 duplicate records in the RBDM. If the duplicate records are excluded, there were 434,467 birth records in the MDC and 404,743 birth records in the RBDM. The analysis is based only on NSW residents. In the MDC, 4,614 records included 4,521 residents of other states and territories; 93 records that did not state place of residence were excluded from the analysis. In the RBDM, 4,963 records included 4,338 residents of other states and territories; 625 records that did not state place of residence were excluded from the analysis. The missing rate in value of living place in RBDM (0.15%) was significantly higher than MDC (0.02%) p < 0.01.

The study was approved by the NSW Population & Health Services Research Ethics Committee, the Human Research Ethics Committees of University of Sydney and University of New South Wales, and the Aboriginal Health and Medical Research Council. Identifying information such as name, address, date of birth and gender obtained from the MDC baby and RBDM birth datasets is included in the Master Linkage Key which is constructed by the Centre for Health Record Linkage (CHeReL).

CHeReL performed the data linkage using probabilistic record linkage methods and ChoiceMaker software (refer http://www.cherel.org.au). At the completion of the process each record in the Master Linkage Key was assigned a record identification number and a Master Linkage Key person ID (Project Person Number (PPN)) to allow linked records for the same individual to be identified and extracted. Linkage quality was assessed with the use of a random sample of 1,000 PPNs in which the false positive rate of the linkage was 0.4% and false negative less than 0.1%.

Descriptive statistics and cross-tabulations were generated for registration rates and proportions. Logistic regression model (enter) was used to explore the factors associated with registration rates. The factors entered into the model included being Indigenous, maternal age, mother’s country of birth, remoteness of living area, birthweight, the quarter of the year in which the baby was born, parity, plurality and neonatal and postneonatal death. The odds ratios (ORs) for the explanatory variables are presented. Some babies had more than one record in the data collections. The duplicate records were identified and excluded before merging the databases.

### Definitions

Maternal Indigenous status: women who have given birth who identify themselves to be of Aboriginal and/or Torres Strait Islander origin.

Live birth: the complete expulsion or extraction from its mother of a product of conception, irrespective of the duration of the pregnancy, which, after such separation, breathes or shows any other evidence of life, such as beating of the heart, pulsation of the umbilical cord, or definite movement of voluntary muscles, whether or not the umbilical cord has been cut or the placenta is attached. Each product of such births is considered to be live born (WHO definition) [3].

Neonatal death: death of a liveborn baby within 28 days of birth [4].

Postneonatal death: death of liveborn baby after 28 days and within one year of birth [2].

The risk group included births with one or more of the following conditions: gestational age <37 or >41 weeks, birth weight <2,500 grams, neonatal or postneonatal death, Indigenous ethnicity, maternal age <25 or >39 years and not living in major cities.

The non-risk group included all remaining births with gestational age 37–41 weeks or birth weight ≥2,500 grams or no neonatal or postneonatal death or baby’s mother being non-Indigenous or aged 25–39 years or lived in major cities.

## Results

For NSW residents, there were 427,134 live born babies between 1 January 2001 and 31 December 2005. There were 61 records excluded from the analysis because they were incomplete. The results are based on the eligible 427,073 births.

The overall registration rate between 2001 and 2005 was 91.80% by the end of 2005. There were 35,017 babies (8.20%) who had not been registered by 31 December 2005. The registration rates are described in detail in Table 1.

**Table 1:** Under-reporting of birth registrations for live births in four years after birth, New South Wales, Australia, 2001–2005

	**Year of birth**	**n**	**Year of birth**		**1st year after birth**		**2nd year after birth**		**3rd year after birth**		**4th year after birth**		**Non- registered**	
			**n**	**%**	**n**	**%**	**n**	**%**	**n**	**%**	**n**	**%**		**n**	**%**
Non- risk group*	2001	44,146	37,117	84.08	4,832	10.95	279	0.63	176	0.40	157	0.36		1,585	3.59
	2002	44,803	38,440	85.8	4,520	10.09	312	0.70	149	0.33				1,382	3.08
	2003	45,579	39,256	86.13	4,445	9.75	267	0.59						1,611	3.53
	2004	45,347	39,364	86.81	4,310	9.5								1,673	3.69
	2005	48,358	41,699	86.23										6,659	13.77
	Total	228,233	195,876	85.82	18,107	10.07	858	0.64	325	0.37	157	0.36		12,910	5.66
Risk group**	2001	40,324	31,562	78.27	4,707	11.67	368	0.91	313	0.78	285	0.71		3,089	7.66
	2002	39,806	31,592	79.36	4,396	11.04	449	1.13	283	0.71				3,086	7.75
	2003	39,421	31,286	79.36	4,271	10.83	424	1.08						3,440	8.73
	2004	38,748	30,946	79.86	4,113	10.61								3,689	9.52
	2005	40,541	31,738	78.29										8,803	21.71
	Total	198,840	157,124	79.02	17,487	8.79	1241	0.62	596	0.30	285	0.14		22,107	11.12
Overall	2001	84,470	68,679	81.31	9,539	11.29	647	0.77	489	0.58	442	0.52		4,674	5.53
	2002	84,609	70,032	82.77	8,916	10.54	761	0.90	432	0.51				4,468	5.28
	2003	85,000	70,542	82.99	8,716	10.25	691	0.81						5,051	5.94
	2004	84,095	70,310	83.61	8,423	10.02								5,362	6.38
	2005	88,899	73,437	82.61										15,462	17.39
	Total	427,073	353,000	82.66	35,594	10.53	2,099	0.83	921	0.54	442	0.52		35,017	8.20

*Non-risk group: non-Indigenous, maternal age 25–39, gestational age 37–41 weeks, no neonatal or postneonatal death, birthweight ≥2,500 grams and living in major cities.

**Risk group has one or more conditions: Indigenous, maternal age <25 or >39, gestational age <37 or >41 weeks, neonatal or postneonatal death, low birthweight (<2,500 grams), not living in major cities.

Missing cases: 61 records which did not state the above conditions are excluded from the analysis.

The majority of births (82.66%) were registered in the same year of birth, and 93.19% of births were registered by the end of the first year after birth. Babies from the risk groups accounted for 46.54% (198,761) of all live births, but accounted for 63.09% (22,091) of the non-registered births.

**Figure 1:**
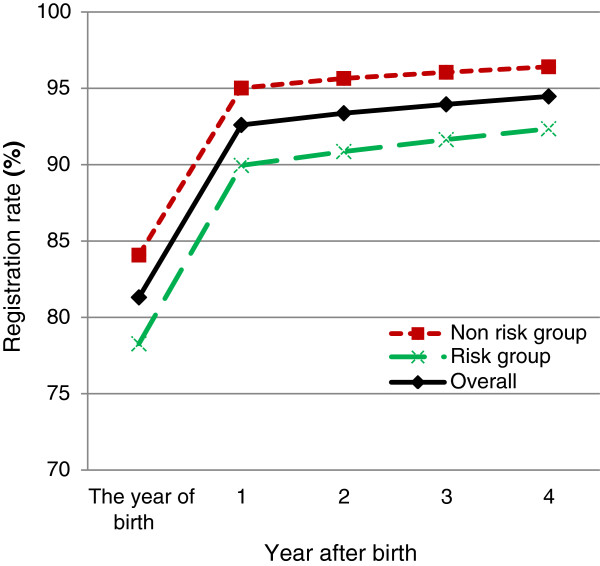
Cumulative birth registration rates (%) of births in 2001, New South Wales, Australia.

**Figure 2:**
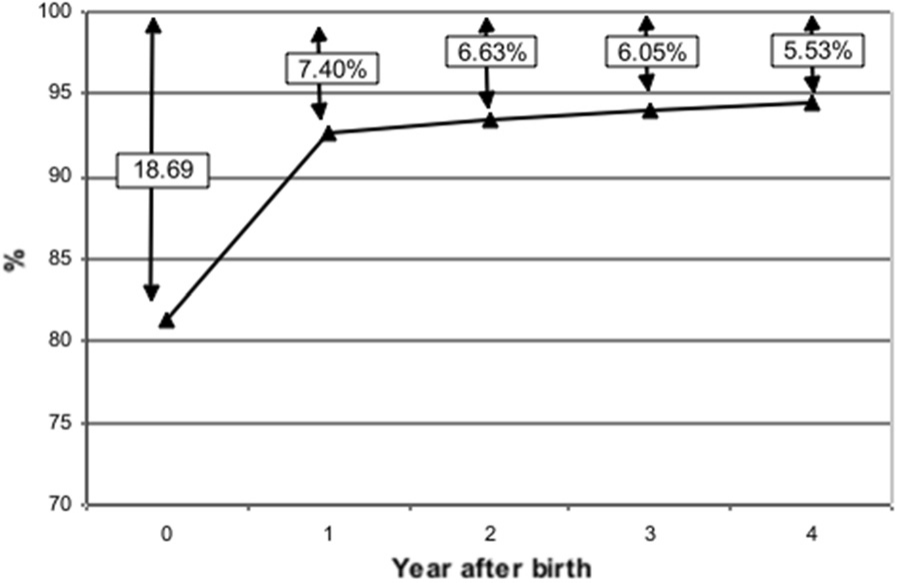
Non-registration rates (%) of births in 2001, New South Wales, Australia.

The births in 2001 had the longest follow-up time. The cumulative birth registration rates of babies born in 2001 are shown in Figures 1 and 2.

The registration rates in the non-risk group were higher than in the risk group. The differences in the 2001 births were 5.81% in the year of birth, 5.07% in the first, 4.79% in the second, 4.41% in the third and 4.06% in the fourth year after birth.

For the births in 2001, there were 4,674 births that were not registered. The non-registration rate in the risk group was 4.06% higher than in the non-risk group (7.66% − 3.60%). The difference was attributed to risk factors and accounted for 53.00% (1,636) of the non-registered births in the risk group. The risk factors can explain 35.00% (1,636) of non-registered births in 2001(1,636/4,674). The rest of the non-registrations (3,038), accounting for 65.00% of the non-registered births, cannot be explained by this data.

Table 2 shows the registration rates and crude OR of risk factors. Factors that were significantly associated with non-registration included: neonatal or postneonatal birth, being Indigenous, maternal age <25 or >39 years, gestational age <37 or >41 weeks, low birthweight, not born in the first quarter of year, mother had previous pregnancies, and living in remote areas. Mothers who were born overseas were more likely to register their births than those born in Australia. Plurality was not statistically associated with non-registration by the crude OR. The year of birth was included in the model as a controlled factor.

**Table 2:** Rates and crude odds ratio for factors associated with non-registration, New South Wales, Australia, 2001–2005 (n = 427,073)

			**Registered**		**Non-registered**				
		**n**	**n1**	**%**	**n2**	**%**	**Crude OR**^**a**^	**95% CI**	
Indigenous status*	Indigenous	14,055	10,023	71.31	4,032	28.69	4.96	4.77	5.16
	Non- indigenous	413,009	382,031	92.50	30,978	7.50	1		
	Missing	9	2	22.22	7	77.78			
	Total	427,073	392,056	91.80	35,017	8.20			
Maternal age*	25–39	332,866	310,270	93.21	22,596	6.79	1		
	<25 or >39	94,193	81,786	86.83	12,407	13.17	2.08	2.04	2.13
	Total	427,059	392,056	91.80	35,003	8.20			
Birthweight (grams)*	≥2500g	401,750	370,078	92.12	31,672	7.88	1		
	<2500g	25,310	21,973	86.82	3,337	13.18	1.78	1.71	1.84
	Missing	13	5	38.46	8	61.54			
	Total	427,073	392,056	91.80	35,017	8.20			
Plurality	Singleton	413,464	379,569	91.80	33,895	8.20	1		
	Multiple	13,609	12,487	91.76	1,122	8.24	1.01	0.95	1.07
	Total	427,073	392,056	91.80	35,017	8.20			
Quarters*^b^	First	105,448	99,499	94.36	5,949	5.64	1		
	Second	105,777	99,522	94.09	6,255	5.91	1.05	1.01	1.09
	Third	109,978	102,812	93.48	7,166	6.52	1.17	1.13	1.21
	Fourth	83,746	78,118	93.28	5,628	6.72	1.20	1.16	1.25
	Total	404,949	379,951	93.83	24,998	6.17			
Number of previous pregnancies*	0	178,720	168,657	94.37	10,063	5.63	1		
	1	143,915	132,948	92.38	10,967	7.62	1.38	1.34	1.42
	2	65,243	58,267	89.31	6,976	10.69	2.01	1.94	2.07
	3	23,702	20,087	84.75	3,615	15.25	3.02	2.90	3.14
	4+	15,215	11,841	77.82	3,374	22.18	4.78	4.57	4.99
	Missing	278	256	92.09	22	7.91			
	Total	427,073	392,056	91.80	35,017	8.20			
Remoteness*	Major cities	321,503	299,357	93.11	22,146	6.89	1		
	Other areas	105,570	92,699	87.81	12,871	12.19	1.88	1.83	1.92
	Total	427,073	392,056	91.80	35,017	8.20			
Mother’s country of birth*	Australia	301,335	278,314	92.36	23,021	7.64	1		
	Other countries	119,776	113,700	94.93	6,076	5.07	0.65	0.63	0.67
	Missing^c^	5,962	42	0.70	5,920	99.30			
	Total	427,073	392,056	91.80	35,017	8.20			
Neonatal or postneonatal death	No	425,870	391,172	91.85	34,698	8.15	1		
	Yes	1,203	884	73.48	319	26.52	4.07	3.58	4.62
	Total	427,073	392,056	91.80	35,017	8.20			
Year of birth	2001	84,470	79,796	94.47	4,674	5.53	1		
	2002	84,609	80,141	94.72	4,468	5.28	0.95	0.91	0.99
	2003	85,000	79,949	94.06	5,051	5.94	1.08	1.03	1.12
	2004	84,095	78733	93.62	5,362	6.38	1.16	1.12	1.21
	2005	88,899	73,437	82.61	15,462	17.39	3.59	3.47	3.72
	Total	427,073	392,056	91.80	35,017	8.20			

^a^ Crude odds ratio (OR).

* Crude odds ratio is significantly different.

^b^ The data from October to December 2005 were excluded because of the interval between birth and registration.

^c^ The missing records of mother’s country of birth were mainly missing from the MDC in 2001 and 2002.

Table 3 shows the adjusted odds ratio of factors associated with non-registration of births. In addition to the nine factors which are significantly associated with non-registration of births in Table 2, multiple births were more likely to be registered compared with singleton births after controlling the nine factors.

**Table 3:** Adjusted odds ratio for factors associated with non-registration, New South Wales, Australia, 2001–2005 (n = 398,730*)

**Factors**	**Values**	**n**	**Adjusted OR**	**95% CI**	
Indigenous status*	Indigenous	12,626	3.26	3.10	3.43
	Non-Indigenous	386,104	1		
Maternal age*	25–39	311,542	1.00		
	<25 or >39	87,188	2.81	2.72	2.90
Birthweight (grams)*	≥2500g	375,296	1		
	<2500g	23,434	1.79	1.69	1.90
Plurality	Single	385,990	1		
	Plural	12,740	0.84	0.76	0.92
Quarters*^b^	First	103,861	1		
	Second	104,183	1.08	1.03	1.12
	Third	108,516	1.30	1.24	1.35
	Fourth	82,170	1.56	1.49	1.64
Number of previous pregnancies*	0	167,942	1		
	1	134,661	1.85	1.78	1.93
	2	60,543	3.06	2.92	3.20
	3	21,751	4.71	4.46	4.98
	4+	13,833	7.29	6.87	7.73
Remoteness*	Major cities	300,897	1		
	Other areas	97,833	1.57	1.52	1.63
Mother’s country of birth*	Australia	285,335	1		
	Other countries	113,395	0.72	0.69	0.75
Neonatal or postneonatal death	No	397,663	1		
	Yes	1,067	3.84	3.23	4.57
Year of birth	2001	80,050	1		
	2002	82,985	8.96	7.98	10.06
	2003	84,932	16.08	14.36	18.00
	2004	84,039	17.39	15.54	19.47
	2005	66,724	25.14	22.44	28.16

*Significantly different.

^b^ The data from October to December 2005 were excluded because of the interval between birth and registration.

## Discussion

Birth data are an essential source of information for governments, researchers and the community. The accuracy and ascertainment of births and registration data are important for planning and research. Birth registration, a state administration’s official record of a baby’s birth, is also important for individuals who use a birth certificate to prove age, parentage and citizenship.

Registration rates differ by country and area. The *Population and development review* published by UNICEF in 1998 reported that the registration rates were 99.8% in Europe, 98.9% in the Americas, 98.8% in Central Asia, 98.1% in the Middle East and North Africa, 90.4% in Sub-Saharan Africa and 76.5% in East/South Asia and the Pacific [5,6]. Industrialised nations registered nearly all their children while developing countries had a lower birth registration rate [5,6]. A cohort study of 766 births between 1985 and 1987 in a rural area of Korea showed that the registration rate was 75.2% within six months and 77.5% within two years [7]. Disparities also exist within countries. In Pakistan, for example, 88% of children born in the Punjab province were registered, while in the North-West Frontier Province the rate was only 46% [5]. Turkey’s western region had a registration rate of 84%, compared to 56% in the east [5].

Few studies report Australian birth registration rates and very few countries have attempted to assess the coverage level of registration objectively and thoroughly [5]. Many countries only report estimates of birth registrations [5]. To date, we have found no literature that linked population data is used to report the registration rate. This study shows that the registration rate in five-years period in NSW was only 94.34% (for births in 2001), which is lower than in Europe, the Americas, Central Asia, the Middle East and North Africa, but higher than Sub-Saharan Africa, and East/South Asia and the Pacific [5]. The difference between the results in *Australia’s mothers and babies 2005* and the current study may be attributed to reporting biases, delayed registration and different data collection methods.

The trend of registration rates in *Australia’s mothers and babies over* the years was similar to the current study, except that the registration rate in the year of birth was relatively higher than in the current study (87.3% versus 82.7%) [2]. *Australia’s mothers and babies 2005* included summary data, and the registration rates were calculated by dividing the number of registrations in the RBDM by the number of MDC births in the same year. The births which occurred outside of Australia but were registered should but not be excluded from the summary data. The linked data of the current study show that 5,741 (1.3%) of NSW residents’ births registered between 2001 and 2005 could not be linked to the birth records in the MDC and were excluded from the analysis. Data linkage can overcome this shortcoming in the summary data by excluding the births registered, but cannot be linked to the midwife’s birth records and accurately distinguish between registered and non-registered records. Consequently, the results from the linked population data are more accurate. However, the unlinked 1.3% of births in RBDM could not fully explain the difference of 4.6% (87.3% − 82.7%). This study implies that the registration rates in NSW were lower than the national registration rate.

This study found that the non-registration rate in NSW was 5.53% and associated with neonatal or postneonatal death, being Indigenous, maternal age <25 or >39, gestational age <37 or >41 weeks, low birthweight (<2,500 grams) and not living in major cities. UNICEF’s report (1998) showed that ethnic minorities, babies born at home and mobile populations have lower rates of registration than the general population [5]. In developing countries, cities tend to have higher rates than rural areas because civil registries are centralised [5]. UNICEF also found that as many as 40 million babies in developing countries are unregistered every year. The main reason is that the systems for reporting births in developing countries are not fully developed [5].

The cohort study in Korea showed that the registration rate within the legal due date was lower in mothers under 20 years of age and above 35 years, and in mothers who had only primary education [7]. The study also showed that the registration rate decreased as the birth order increased, and was higher in births that occurred between October and March than births that occurred between April and September [7]. All of the births for seven neonatal deaths in the Korean study were not reported [7].

The ABS publication, *Births, Australia*, shows the percentage of birth years when births were registered [1]. Of the births registered in NSW in 2006, 88.6% were born in the year of birth, 10.1% were born in 2005, 0.7% in 2004, 0.3% in 2003 and 0.1% in 2002 and 2001 [1]. The results suggest that there was a delay in registration. However, the registration rates could not be calculated from the data because they did not include the births that were born in NSW but were non-registered.

Reasons suggested for the interval between the occurrence and the registration of a birth included delays by the parents in submitting a completed form to the registry or delays by the registry in processing the birth. Hospitals and birth clinics notify state registries of recent births on a regular basis. For those births known to a registry that have not been registered within a prescribed time period, a reminder letter is sent to the parents of the child as a follow-up. Under the *Births, Deaths and Marriages Registration Act 1995* all births in NSW are to be registered within 60 days of a child’s birth [8-10]. The Registry processes the Birth Registration Statement within 14 days of receiving the application. The 74 days (60 days plus 14 days) account for 21% of a year. The registration rate in the year of occurrence of the birth should be 79% or more. This study showed that 83% of births were registered in the year of occurrence. This implies that most parents adhered to the time requirement for registration of births. On the other hand, those who did not register their babies within the time requirement were less likely to register in the following years.

The Baby Bonus is a non-income tested lump sum payment for each child born (including stillborn) or adopted by Australian families. Parents were not required to formally register the birth of their child as a condition of receiving the Baby Bonus for births prior to 1 July 2007 [11]. This might be a reason for birth non-registrations. Since 1 July 2007, the birth registration rate should have improved, not including the stillborn, adopted and those born outside of NSW, because this requirement does not apply to parents whose child is stillborn, adopted or born outside of NSW [11].

Indigenous births were less likely to be registered. The average interval between the occurrence and registration of the birth was longer in Indigenous births (6.4 months in 2006) than in the general population (2.2 months in 2005) [1,12]. The average interval between the occurrence and registration of Indigenous births varied across the states and territories. Western Australia and Queensland recorded the largest average intervals (10.4 and 9.7 months respectively) in 2006, and the Northern Territory and Tasmania recorded the lowest average intervals (1.4 and 2.5 months respectively) [1,12].

The fee charged for registration and birth certificates are economic barriers for birth registration and UNICEF recommends free birth registration and birth certificates [13]. In NSW, birth registration is free but a fee is charged for the birth certificate; this fee increases regularly. Between 1 December 2007 and 31 November 2008 a birth certificate cost AUD36.00 (about USD 37.66, EUR 29.03), and AUD42.00 (about USD 43.94, EUR 33.87) since 1 December 2008 [14]. According to UNICEF, a birth certificate only cost AUD29.00 (about USD 30.34, EUR 23.38) in NSW in 2002 [13]. The impact of the fee increase of a birth certificate on the registration rate in NSW needs to be investigated.

Approximately 3.60% of babies in the non-risk group were non-registered; this cannot be explained by delayed registration or the risk factors mentioned above. A further study, in which parents of non-registered babies are interviewed, is necessary to identify other barriers to registration.

The Midwives Data Collection (MDC) is a population-based surveillance system covering all births including live births and stillbirths in NSW public and private hospitals, as well as homebirths. In 1992, the MDC became a statutory data collection under the NSW *Public Health Act 1991*. It received notifications of all births which occurred in NSW [4]. The attending midwife or doctor completes a notification form (or its electronic equivalent) when a birth occurs. The completed forms are sent to the NSW Department of Health where they are checked and compiled into the MDC database. The RBDM is a database of birth registrations. Under the *Births, Deaths and Marriages Registration Act 1995*, all babies in NSW must be registered within 60 days of birth. The hospitals in which the birth occurs, or the child and mother are taken to within 24 hours of the birth, supply a birth registration statement (BRS) to parents and a notification to the RBDM. If the birth occurs at home or in a location other than a hospital with a registered doctor or registered midwife in attendance, the registered doctor or midwife advises the RBDM of the birth and also provides the mother with a BRS. If the birth occurs in a location other than a hospital without a registered doctor or midwife in attendance, and the child is not taken to a hospital within 24 hours of the birth, the mother should call the RBDM and provide her name and home address, and names and addresses of two independent witnesses who saw the birth. Statutory declarations are then posted to the witnesses. Once they are signed and returned to the RBDM, a BRS is posted to mothers [8]. If 100% of births in NSW were registered, the number of births in the RBDM would be slightly higher than in the MDC because the RBDM includes births notified other than by medical staff. However, the RBDM reports fewer births than the MDC [2]. For the current study, the births records in MDC were more complete and used as a ‘gold standard’.

There were several limitations in this study which may have impacted its results. The analysis of factors was limited to the variables available in the linked data. Birth place, marital status and socio-economic level should be included in future studies. On the other hand, the interval from birth to registration cannot be calculated because the month of registration is not available in this study. However, the birth registration rates in this study imply that the median was less than one year. In some underdeveloped areas or countries, many children are registered later in life, for example when they enrol in school [5]. If the follow-up period in this study was extended to 10 years after birth, this factor could be assessed. A survey of the non-registered group is necessary to identify more reasons.

## Conclusion

The majority of births (82.66%) were registered in the same year of birth and 93.19% of births were registered in the first year after birth. There were 4.92% of NSW births that were not registered by the fourth year. Around one-third of birth non-registrations in NSW could be explained by the following factors: neonatal or postneonatal death, Indigenous status, maternal age <25 or >39, gestational age <37 or >41 weeks, low birthweight (<2500 grams) and not living in major cities. Meanwhile, the remainder (65%) of non-registrations could not be explained.

## Competing interests

The authors declare that they have no competing interests.

## Authors’ contributions

FX participated in the study design, data analysis and paper writing. LH participated in the study design. MPA participated in diagnoses grouping. EAS participated in the study design and coordinated and supervised the study. All authors read, revised and approved the final version of the manuscript.

## Pre-publication history

The pre-publication history for this paper can be accessed here:

http://www.biomedcentral.com/1471-2393/12/147/prepub
